# RNAMotifProfile: a graph-based approach to build RNA structural motif profiles

**DOI:** 10.1093/nargab/lqae128

**Published:** 2024-09-26

**Authors:** Md Mahfuzur Rahaman, Shaojie Zhang

**Affiliations:** Department of Computer Science, University of Central Florida, 4328 Scorpius Street, Orlando, FL 32816-2362, USA; Department of Computer Science, University of Central Florida, 4328 Scorpius Street, Orlando, FL 32816-2362, USA

## Abstract

RNA structural motifs are the recurrent segments in RNA three-dimensional structures that play a crucial role in the functional diversity of RNAs. Understanding the similarities and variations within these recurrent motif groups is essential for gaining insights into RNA structure and function. While recurrent structural motifs are generally assumed to be composed of the same isosteric base interactions, this consistent pattern is not observed across all examples of these motifs. Existing methods for analyzing and comparing RNA structural motifs may overlook variations in base interactions and associated nucleotides. RNAMotifProfile is a novel profile-to-profile alignment algorithm that generates a comprehensive profile from a group of structural motifs, incorporating all base interactions and associated nucleotides at each position. By structurally aligning input motif instances using a guide-tree-based approach, RNAMotifProfile captures the similarities and variations within recurrent motif groups. Additionally, RNAMotifProfile can function as a motif search tool, enabling the identification of instances of a specific motif family by searching with the corresponding profile. The ability to generate accurate and comprehensive profiles for RNA structural motif families, and to search for these motifs, facilitates a deeper understanding of RNA structure–function relationships and potential applications in RNA engineering and therapeutic design.

## Introduction

The three-dimensional (3D) structures of non-coding RNAs (ncRNAs) are becoming one of the main focuses of recent studies due to their participation in many biological functions (1–4). The 3D structures are also found more conserved than their sequences over the course of evolution (5). To comprehensively analyze the 3D RNA structures, they can be examined through various structural elements, including loops, helices, pseudoknots and long-range tertiary motifs. The loop regions, in particular, play a crucial role and are further categorized as hairpin loops, internal loops and multi-loops (6). These loop regions are modular, recurrent and structured by conserved non-Watson–Crick base pairs (7). These recurrent loop regions are generally referred to as structural motifs (8). The term *structural motif* can be defined from different aspects. However, as RNA structural motifs typically comprise an entire hairpin or internal loop to perform their structural recognition, interaction or enzymatic role (8), we will use the term *RNA structural motif* to represent the structural loop motifs throughout this paper. Therefore, the collections of these motifs will be mentioned as structural motif families. Among these motif families, a few of them are well studied and known for their structures, such as kink-turn (9), sarcin–ricin (10) and E-loop (11). Several studies (12,13) focus on smaller recurrences and categorize motifs such as A-minors (14), ribose zippers (15) and coaxial helices (16). These motifs are comparatively compact but contain structural variations and correlations among them.

**Figure 1.**
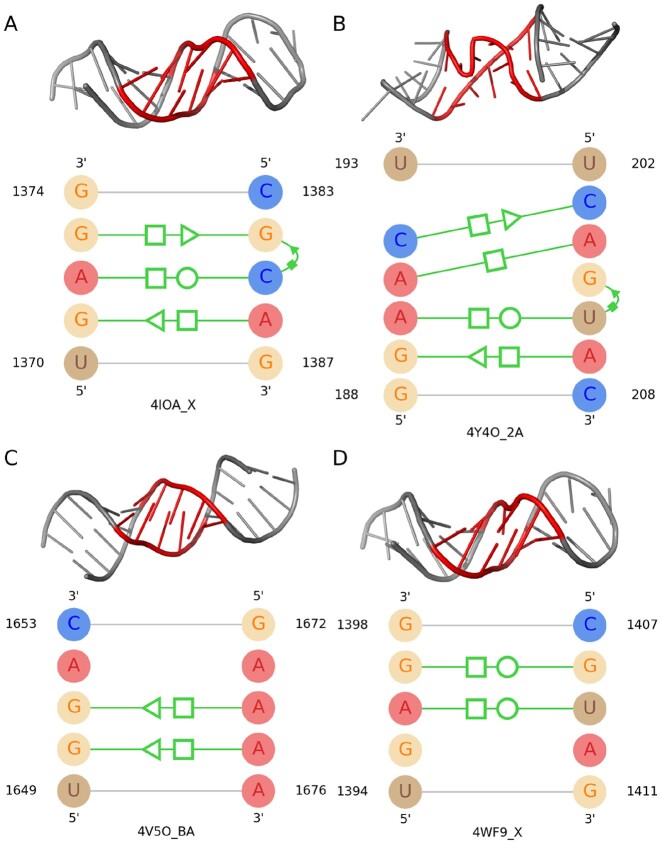
An illustration of the interaction (only base-pairing interactions are shown) similarities among the instances of different motif families and the variations in the instances of the same motif family. In each part of this figure, the top one is the 3D structure of a motif with its base-pairing interactions at the bottom. (**A**) A motif instance from E-loop, (**B**) a motif instance from sarcin–ricin and (**C**, **D**) two motif instances from E-loop. The first two instances show the common interactions between different motif families, while the last two show the variation of interactions in the same motif family. The proposed notations in the work by Leontis *et al.* (23) are used to represent the non-canonical base pairings in this and the following figures.

There exist several studies that work to find the recurrent structures of well-known motifs based on their structural similarity, such as RNAMotifScan (17), RNAMotifScanX (18) and FR3D (19). Moreover, there are several studies (20,21) that find the recurrent structures by clustering motif instances using RNAMotifScan or FR3D alignments. In these studies, the 3D structures of motif instances from the same family are generally characterized by a common set of isosteric base interactions, though this consistent pattern is not observed without exception across all instances. Based on the variations they have in their 3D structures, each motif family can be distributed into multiple subfamilies. Besides, the structures of different motif families might share similar interactions, which introduces confusion to the process of identifying instances of those particular motif families. One such example is shown in Figure 1. Although the top left instance of this figure is from the E-loop motif family and the top right one is from sarcin–ricin, they share four base-pairing interactions. On the contrary, even though both the bottom two instances of Figure 1 are from the E-loop motif family, they do not have any base pairs in common. RNAMotifComp (22) analyzes multiple motif families to identify similar instances across families based on their base interactions and 3D formations. These similarities and variations inside motif families made the analysis of RNA structural motifs more complex and challenging.

RNAMotifContrast (24) mostly compares and contrasts the similarities as well as variations in the motif instances of well-known motif families based on their structures. It also introduces the concept of subfamilies by identifying the variations among the motif instances of the same motif family. Although RNAMotifContrast successfully shows the comparison of instances from a motif family by superimposing them together, it does not analyze the statistical data of all base interactions throughout the instances of a specific family, subfamily or group. To analyze the variations and characteristic interactions of a motif group, it is important to have weighted data to work with, and here comes the urge to find a method to build a structural profile for a group of motif instances that could be of a family or subfamily. The profile enables the identification of conserved structural features that can provide insights into the shared functional characteristics of the motif group. The profile can be used further to analyze the potential binding sites or interactions with other biomolecules within that motif group, facilitating the understanding of their functional roles.

There are studies that use sequence alignment or secondary structure alignment to generate profiles and predict new or recurrent RNA structures. For instance, Gautheret and Lambert (25) used sequence alignment with secondary structure information of an RNA to define RNA signatures and found occurrences in sequence databases. Hochsmann *et al.* (26) utilized a tree alignment model to compute multiple alignments of RNA secondary structures. Soulé *et al.* (27) described an approach to identify recurrent interaction networks by finding the hierarchical organization of RNA secondary structure elements through maximal common subgraph matching. While these studies have contributed to the understanding of RNA structures, they primarily focus on secondary structures, which may not accurately represent the actual 3D conformations adopted by RNAs in their native environments. Furthermore, efficient and accurate computational methods are often required to predict RNA secondary structures reliably. This limitation highlights the need for approaches that can directly analyze and compare RNA 3D structural motifs, which are more conserved and functionally relevant than secondary structures.

In this paper, we have proposed a method to create the structural profile of a group of RNA structural motif instances. Our main goal is to preserve the sequence and interaction data for all the instances in a single and easily interpretable data structure that can be used to get significant insights from that group of motif instances. The structural profile represents the variability of structural properties in a particular RNA motif group along with their sequences. An RNA motif profile should incorporate information such as base-pairing interactions, base-stacking interactions and nucleotide sequences from a group of RNA structural motif instances into a single profile that could be a vital resource in analyzing those instances. The profile data can also be utilized to search or predict other similar motif instances from an unknown dataset. A profile also exhibits the conservation level of various information throughout that motif group. We designed a clique-based profile-to-profile alignment algorithm to build a profile of a set of input motif instances. First, each motif instance will be considered as an individual profile. Then, following a guide-tree-based profile-to-profile alignment approach, two profiles will be aligned and merged until one single profile exists as output. To visualize the profile built from a group of input motifs instances, we created a 2D image for each profile accommodating multiple nucleotides in one position and multiple base interactions between two nucleotide positions. Finally, we have employed the profile-to-profile alignment algorithm to search for similar structures throughout an RNA chain.

## Materials and methods

**Figure 2.**
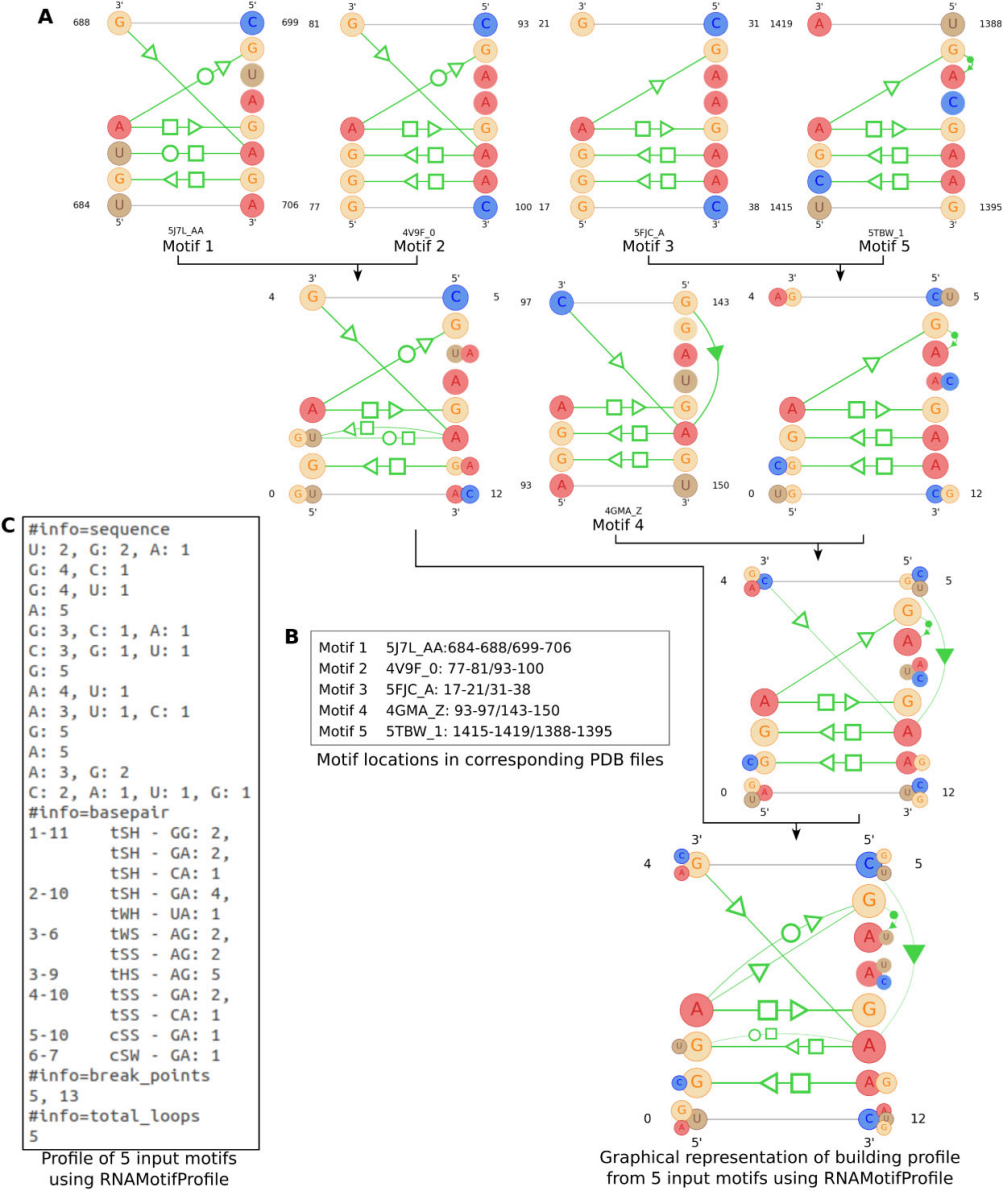
The profile-to-profile alignment steps with five kink-turn motif instances. (**A**) Steps to build a profile from a set of input motif instances. In each step, the best-aligned profile pair is merged into a new profile. The process continues until there is one resulting profile. (**B**) The PDB locations of the set of motif instances are provided as input. (**C**) The profile output is in text format (base-stacking interactions are not shown due to space constraints).

The profile-to-profile alignment in RNAMotifProfile is inspired by the structural motif alignment algorithm from RNAMotifScanX. However, RNAMotifProfile differs significantly from RNAMotifScanX in its approach and capabilities. While RNAMotifScanX aligns one structural motif with another and generates an alignment score to represent the quality of the alignment, it performs a local alignment focused solely on matching base interactions. In contrast, RNAMotifProfile aligns one structural profile to another, not only considering the local alignment of base interaction profiles but also performing sequence profile alignment for the remaining partial unaligned regions to generate a complete alignment of the two profiles. In RNAMotifProfile, we take a group of RNA structural motif instances as input, which could be from a well-known motif family or subfamily. The nucleotide sequences and the coordinates of atoms for each motif instance are then collected from the associated PDB and FASTA files. For each PDB file, base interaction annotation files are generated by using the annotation tools or obtained from the corresponding websites. These base interaction annotation files are then used to create individual motif files for each motif instance by incorporating base interaction information for the particular motif. Each motif is then converted to represent an individual profile using the same data structure that we designed for profiles. By following a guide-tree approach, these profiles are aligned together, with the best-aligned profile pairs being aligned and merged in each step until a single profile remains. This profile data are then utilized to draw an image that helps to visualize the prevalence of different interactions in each location. Five instances of the kink-turn motif family are used to demonstrate the steps in Figure 2 and the details are discussed in the following sections.

### Notations and basic definitions

Let *X* and *Y* be the two structural motif profiles that are being aligned using RNAMotifProfile. The sequence profile of *X* is denoted by $S_P^X$ and the length is $|S_P^X|$. The *i*th nucleotide profile of $S_P^X$ is denoted by $S_P^X[i]$ and a sub-portion of the sequence profile that begins with $S_P^X[i]$ and ends with $S_P^X[j]$ will be denoted by $S_P^X[i, j]$, inclusively. Let ${\rm BP}_P^X(i, j)$ denote the base-pairing profile between $S_P^X[i]$ and $S_P^X[j]$, and similarly, ${\rm STK}_P^X(i, j)$ denote the base-stacking profile between $S_P^X[i]$ and $S_P^X[j]$. As each profile contains a combined information of more than one motif instance, we wanted to keep track of that number and denoted the motif count for profile *X* as MC^*X*^. We also keep track of the break points in each profile and denote it as BrP^*X*^, which is a list of indices of break points in motif profile *X*. In the context of RNA structural motifs, we define break points as the nucleotide positions where the base-pairing interactions are interrupted, marking the beginning and end of loop regions. The number of break points in a motif instance corresponds to the number of segments that form the loop (hairpin, internal or multi-loop). A hairpin loop motif contains a single break point, as it consists of one continuous loop region flanked by base-paired stems. An internal loop motif contains two break points, one at the end of each of the two loop segments separating the two base-paired stem segments. A multi-loop motif contains three or more break points, with each break point marking the junction between a stem segment and a loop region. Therefore, the number of break points in a motif instance directly relates to the type and complexity of the loop structure, with more break points indicating a more complex multi-loop configuration.

The goal of the RNAMotifProfile algorithm is to globally align two profiles and merge their structural information together to produce a new profile. To do so, RNAMotifProfile uses an optimal local alignment algorithm to find the anchors of base interaction profiles first and then extend the alignment by performing sequence profile alignment for the unaligned segments. For example, from the local alignment algorithm, if we find $S_P^X[i, j]$ is aligned with $S_P^Y[k, l]$, given $|S_P^X| = m$, $|S_P^Y| = n$, 1 < *i* < *j* < *m*, 1 < *k* < *l* < *n*, then it will be used as an anchor and $S_P^X[1, i-1]$ will be aligned with $S_P^Y[1, k-1]$ and $S_P^X[j+1, m]$ will be aligned with $S_P^Y[l+1, n]$ based on their sequence profile to get the total alignment of the two input profiles. Finally, the aligned interactions along with their nucleotide data are merged together to have a single profile as output. The performance of an alignment is measured by an alignment score that is calculated based on matching base-pairing, base-stacking and sequence profiles. We have used three scoring matrices to compare base-pairing interactions, base-stacking interactions and nucleotides. The base-pairing interaction scoring matrix is derived from RNA base-pair isostericity (23,28), employing 18 isosteric matrices to account for both directions of isosteric base pairs. The scoring matrices for base-stacking interactions and nucleotide substitution are adopted from RNAMotifScanX. Additionally, we have used a weighted scoring formula to calculate the alignment score of two candidate profiles.

### Collect structure and annotation data from PDB files

For the input motif instances (discussed in the ‘Results’ section), we downloaded the corresponding PDB and FASTA files from the PDB database. For each of these PDB data, we collected FR3D (19) annotations from the RNA Structure Atlas website (29) and generated DSSR (30) annotations using the DSSR annotation tool v1.7.8-2018sep01. However, we have implemented the annotation collection part in such a way that, if the DSSR tool does not exist in the working computer, the annotations will be downloaded from the DSSR website (31). These two annotation results are then merged together to get more accurate base interaction information for each location. While merging annotation data for a single PDB from two annotation sources, it is possible to encounter different annotations for the same pair of bases. These conflicts are addressed by considering the likelihood of possible interactions between a pair of nucleotides. We counted the interaction frequencies for all possible nucleotide pairs of the RNAs collected from the Representative PDB list (32) release 3.320 at a resolution of 4.0 Å. The interaction with higher frequency is considered to be more likely to be present between a pair of nucleotides. Therefore, the conflicts are resolved by taking the interaction that is observed more throughout all RNAs. By utilizing these base interaction annotation data, we have prepared individual loop files from each input motif instance that contain the sequence, base-pairing and base-stacking data of that particular instance. Additionally, we create a partial PDB file for each motif to keep track of the corresponding coordinates of each motif instance. We use these partial PDB files to visualize their 3D structure in PyMOL.

### Convert motif instances to individual profiles

As RNAMotifProfile is an algorithm for profile-to-profile alignment, it is required to convert each input motif instance to an individual profile. In order to gather multiple nucleotide and base interaction data in a single index, we utilized the hash table data structure. We store multiple nucleotides of a single index with their number of occurrences by maintaining a hash table for each index. We use separate hash tables for base-pairing and base-stacking interactions, respectively. We also keep track of the total number of motif instances used to generate a profile. Additionally, as the motif instances can be of multiple segments, we store the break point information in the generated profile as well. While converting a motif to its profile form, the number of occurrences of all the nucleotides and base interactions remains 1 and no indices contain more than one nucleotide or interaction.

### Profile-to-profile alignment

As mentioned earlier, RNAMotifProfile uses a profile-to-profile alignment algorithm and generates an alignment score to show the alignment quality. Higher alignment scores represent better alignments, while the lower score means poor alignment results. The brute-force comparison of each interaction profile with another will require exponential time, so it was necessary to find an efficient way to compare and find matches between two structural profiles. RNAMotifScanX earned a better runtime performance by utilizing a clique-based graph alignment algorithm (33). We have employed the same algorithm for RNAMotifProfile, but instead of matching one interaction to the other, we have to come up with an idea to match one interaction profile to the other. To do so, we introduced several formulas to calculate the weighted score while matching two profiles. By summing up these weighted scores for base-pairing, base-stacking and nucleotide profiles, we calculated the alignment score between two motif profiles. The alignment score of two profiles is denoted by *S*(*P*), and the formula is defined as


(1) \begin{eqnarray*} S(P) = \sum _{i,j} \lbrace S^{{\rm BP}} (i, j) + S^{{\rm STK}} (i, j) + S^N (i) + S^N (j)\rbrace , \end{eqnarray*}

where ${\rm S}^{BP}(i, j)$ represents the weighted score of matching ${\rm BP}_P^X(i, j)$ with ${\rm BP}_P^Y(i, j)$. Similarly, ${\rm S}^{STK}(i, j)$ represents the weighted score of matching ${\rm STK}_P^X(i, j)$ with ${\rm STK}_P^Y(i, j)$ and *S*^*N*^(*i*) represents the weighted score of matching nucleotide profile $S_P^X[i]$ with $S_P^Y[i]$. If there are *m* base-pairing interactions in ${\rm BP}_P^X(i, j)$ and *n* base-pairing interactions in ${\rm BP}_P^Y(i, j)$, the weighted score between ${\rm BP}_P^X(i, j)$ and ${\rm BP}_P^Y(i, j)$ is calculated using the following formula:


(2) \begin{eqnarray*} S^{{\rm BP}} (i, j) = \frac{1}{m} \sum _{a=1}^m \frac{1}{n} \sum _{b=1}^n {\rm Count}({\rm BP}_b^Y) \times M({\rm BP}_b^Y, {\rm BP}_a^X) , \end{eqnarray*}

where ${\rm Count}({\rm BP}_b^Y)$ represents the frequency of the *b*th base pair in ${\rm BP}_P^Y(i, j)$ and $M({\rm BP}_b^Y, {\rm BP}_a^X)$ represents the matching score of ${\rm BP}_b^Y$ with ${\rm BP}_a^X$, which is derived from the isosteric base-pairing interaction scoring matrix mentioned earlier. Similarly, the following formula is used to calculate the weighted score between ${\rm STK}_P^X(i, j)$ and ${\rm STK}_P^Y(i, j)$:


(3) \begin{eqnarray*} S^{{\rm STK}} (i, j) = \frac{1}{m} \sum _{a=1}^m \frac{1}{n} \sum _{b=1}^n {\rm Count}({\rm STK}_b^Y) \times M({\rm STK}_b^Y, {\rm STK}_a^X). \nonumber\\ \end{eqnarray*}

The weighted scores for corresponding nucleotide profiles are calculated using the following formula:


(4) \begin{eqnarray*} S^N (i) = \frac{1}{m} \sum _{a=1}^m \frac{1}{n} \sum _{b=1}^n {\rm Count}(S_b^Y) \times M(S_b^Y, S_a^X), \end{eqnarray*}

where ${\rm Count}(S_b^Y)$ is the frequency of the *b*th nucleotide in $S_P^Y[i]$ and $M(S_b^Y, S_a^X)$ denotes the matching score. RNAMotifProfile enumerates all possible compatible combinations of base interaction profiles to find the optimal maximum alignment score.

**Figure 3.**
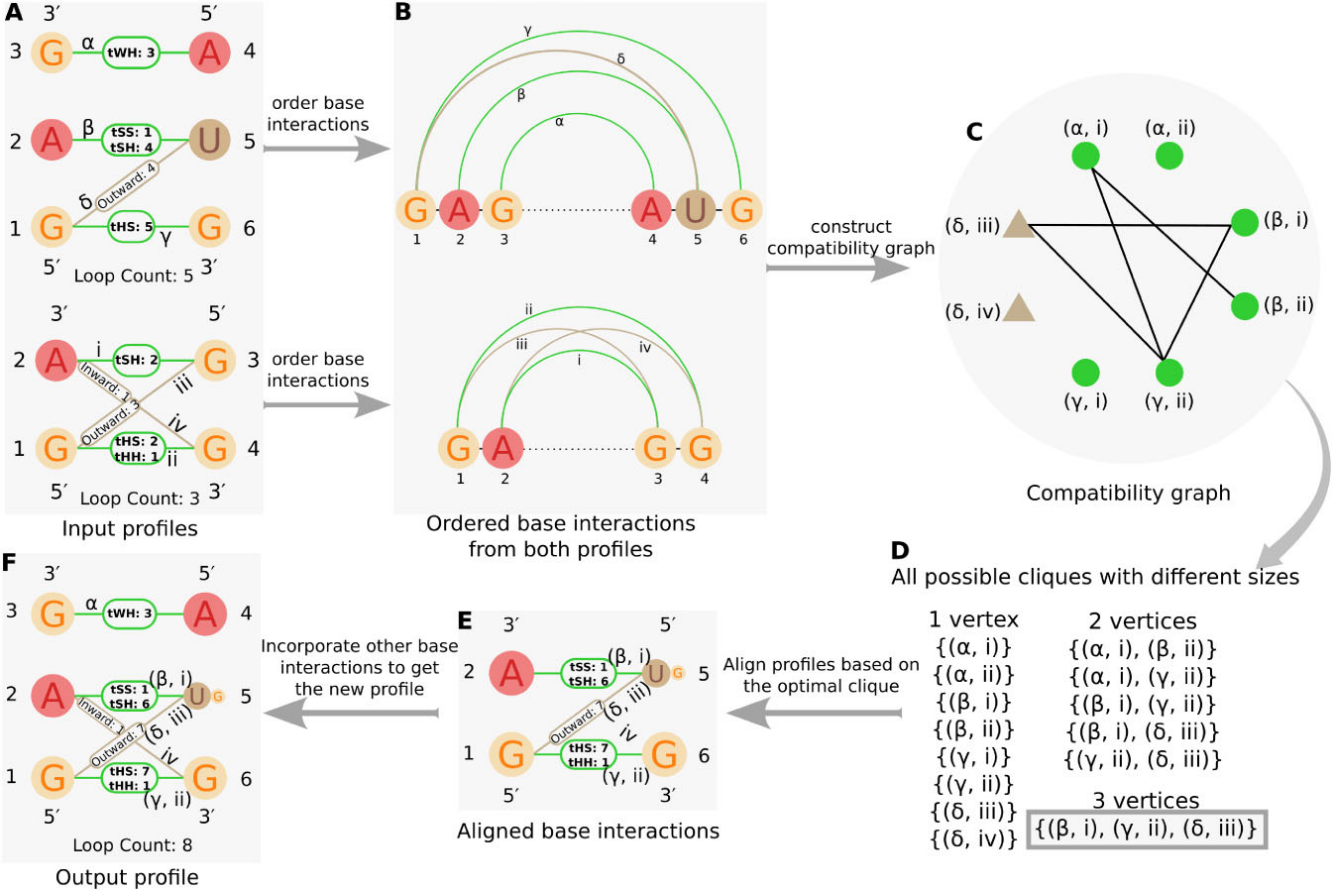
The algorithmic framework of RNAMotifProfile is demonstrated by aligning two artificial motif profiles. (**A**) Two input motif profiles, one from five motif instances and the other from three motif instances. (**B**) Ordered base interactions. (**C**) The compatibility graph is made from the ordered base interactions. (**D**) All possible cliques. (**E**) Aligned base interactions based on maximal clique. (**F**) Output profile from the clique-based alignment after incorporating other nucleotides as base interactions. The output of the algorithm is the optimal alignment between the two input motif profiles in terms of a weighted combination of base-pairing profile, base-stacking profile and sequence profile similarity.

The profile-to-profile alignment algorithm is depicted in Figure 3. First, the base interaction profiles for each nucleotide pair are ordered with the following criteria: ${\rm BP}_P^X (i, j)$ will be placed before ${\rm BP}_P^X (k, l)$ if *i* < *k*, or *i* = *k* and *j* < *l*, assuming the nucleotide profile positions (*i*, *j*, *k*, *l*) are numbered from 5′ end to 3′ end. Then, a compatibility graph is generated by considering six relation groups defined in RNAMotifScanX paper. The relation groups are the following: juxtaposing, juxtaposing with shared nucleotide, crossing, enclosing, enclosing with shared nucleotide (left) and enclosing with shared nucleotide (right). For a detailed explanation and figure of these relation groups, please refer to (18). A base-pair profile matching formed between ${\rm BP}_P^X(i, j)$ and ${\rm BP}_P^X(i^{\prime }, j^{\prime })$ is consistent with the one formed between ${\rm BP}_P^X(k, l)$ and ${\rm BP}_P^X(k^{\prime }, l^{\prime })$, if and only if the relation group in which ${\rm BP}_P^X(i, j)$ and ${\rm BP}_P^X(k, l)$ are classified equivalent to the relation group in which ${\rm BP}_P^X(i^{\prime }, j^{\prime })$ and ${\rm BP}_P^X(k^{\prime }, l^{\prime })$ are classified. Each vertex in the compatibility graph represents a compatible base interaction matching. Obviously, each base-pairing profile is compared with only another base-pairing profile, and the same criteria are maintained for base-stacking interactions. After generating the compatibility graph, all possible cliques are identified with different sizes, and the alignment score is calculated for each combination by utilizing the Bron and Kerbosch algorithm (33). Our goal was to obtain a higher alignment score while ensuring an optimal clique so that we could find expected matching interaction profiles. The runtime of the clique-finding algorithm is directly proportional to the size of the compatibility graph. A higher number of interactions results in more edges, increasing the graph’s complexity. While both base-pairing and base-stacking interactions can be feasibly utilized for smaller motif instances (e.g. hairpin loops), this approach becomes impractical for larger motifs such as internal or multi-loop structures. To optimize the algorithm’s runtime, we implemented a strategy that uses base-pairing interactions as anchors during clique finding, while still considering both base-pairing and base-stacking interactions in alignment score calculations. This approach significantly reduces computational time without compromising the quality of the results. More details are provided in the ‘Computational efficiency of RNAMotifProfile’ section.

We have also employed a branch-and-bound technique, similar to the one used in RNAMotifScanX, to improve the runtime of our algorithm. This approach leverages precalculated maximum alignment scores to enable early termination of unpromising search paths. As we iteratively expand cliques in the compatibility graph, we maintain two key values: a lower bound and an upper bound. The best alignment score achieved so far by existing base interaction matching is stored as the lower bound, while an optimistic forecast of the maximum possible score achievable through future expansions is considered as the upper bound. The lower bound is simply updated with each new best score encountered. For the upper bound, we utilize the graph structure. Given *k* unmatched vertices in set *V*, we know there must be at least *k*(*k* − 1)/2 edges between these vertices to form a clique. By counting the actual edges in *V*, we can determine the maximum number of possible additional matchings. This allows us to compute an upper bound on the potential score improvement. Our algorithm terminates a search path of a maximal optimal clique when its upper bound falls below the current lower bound, as this indicates that continuing along this path cannot yield a better solution than what we have already found. This approach significantly reduces unnecessary computations, especially for less promising alignment paths. Finally, the unaligned base interactions are incorporated accordingly based on their nucleotide positions to the aligned profiles so that we do not lose any structural information of the input instances.

As we have to output a profile by merging multiple profiles and at the same time handle different types of interactions in the same nucleotide location pair, we have utilized a guide-tree-based approach to generate the profile. After gathering all information on each motif, we convert them into profiles. We calculate the profile–profile alignment score for all possible profile pairs and identify one pair with the highest score as the best-aligned profile pair. Then, we take off the two profiles from the profile list, merge them into one and store it in the same list. We repeat this procedure until we end up having one single profile in the list. The procedure is depicted in Figure 2 using an example of five motifs. While generating a profile for a large number (e.g. 20 instances) of motifs, it might take more time to find the best-aligned pair. To compensate for this issue, we distribute the input motif instances into small chunks (e.g. five instances) and follow the same procedure described above to build profiles for each chunk. Finally, profiles built from the chunks are merged using the profile–profile alignment algorithm to get the expected profile. The whole process of distribution into small chunks and merging their profiles is done recursively until we get a single profile. A simplified pseudocode of the profile-building algorithm is provided in the Supplementary Algorithm S1.

While aligning two profiles, it is possible to have better alignment when aligned in different orientations for internal loops and three or more way junction loops (multi-loops). RNAMotifProfile is designed to handle such cases and keep track of their orientation each time two profiles are aligned. For example, let us consider we have an internal loop profile with two segments—the first one is of length 7 starting with index 1 and ending with index 7, and the second one is of length 5 starting with index 8 and ending with index 12 (there will be no index gap between two segments in a profile). Suppose there is a base-pairing profile between two nucleotides indexed in 2 (second index of the first segment) and 10 (third index of the second segment). In a different orientation of the same profile, the length of the strands will flip—the first one will be of length 5 (indices 1–5) and the second one will be of length 7 (indices 6–12). For this orientation, along with the nucleotide indices adjustment, the index for the base-pairing profile will have to be adjusted too. For this example, the base-pairing profile in the second orientation will be between 3 (third index of the first segment) and 7 (second index of the second segment). The edges of all the interactions in the base-pairing profile will have to be reversed as well [e.g. *trans*-Hoogsteen/sugar (*trans*-H/S) interaction will become *trans*-sugar/Hoogsteen (*trans*-S/H)]. The same procedure was followed to generate all possible orientations for multi-loop motifs while building multi-loop motif profiles.

### Representing structural profiles

RNAMotifProfile builds the profiles and provides a text file with .pfl extension as output. This file starts with the sequence profile followed by base-pairing profile, base-stacking profile, break points (for internal and multi-loop motif profiles) and the total number of motifs used to build this profile. In the sequence profile portion, the nucleotide letters along with their observed frequencies throughout the input motifs are provided. It also includes gaps (–), which represent some of the input motifs that do not have a nucleotide in that particular position of the profile. The next part contains base-paring profile where the observed base pairs are listed with their frequencies. It is possible to find the same base pair with more than one different nucleotide pair between two positions. Therefore, the frequencies were counted separately for different nucleotide pairs. For example, if a *trans*-H/S base pair is observed with both G–G and G–A pair, the frequencies were counted separately. The following part contains base-stacking profiles with their frequencies. The break points and the total number of participating loops are added at the end of the profile output. All the nucleotide, base-paring and base-stacking interaction information is sorted based on their frequencies.

Representing such structural profiles using 2D images is quite challenging due to having more than one nucleotide and base-pairing interaction in each location. We have implemented a computational approach to create a graphical visualization of the structural profiles from their text-based representations. We used weighted circles based on their frequencies to represent multiple nucleotides for each location. Also, we have added more than one edge between two nucleotides if there exist multiple base-pairing interactions. The thickness of the edges varies based on their frequencies. It is possible to get an edge between two nucleotide positions while not all nucleotides in those positions participate in the base-pairing interaction. To differentiate such nucleotides, we used a thin border outside of the circles representing the nucleotides that participate in the particular base interaction. Moreover, the base interactions that appeared in less than 10% of the instances and the gaps that appeared in more than 75% of the instances are filtered while drawing the images. This filtering is applied in order to keep the image clean from less significant interactions and gaps. Therefore, even though the graphical representation creates an opportunity to visualize an RNA structural profile, the text output will be more reliable to get accurate information of the profile.

### Searching motifs using profiles

The core algorithm of RNAMotifProfile is the profile-to-profile alignment. By utilizing this algorithm, RNAMotifProfile can easily be applied as a motif search tool. For example, if we have a profile of the kink-turn motif and we would like to search all possible kink-turn motifs from an RNA chain, we can utilize the search feature of RNAMotifProfile. To do so, we take the RNA chain as input and the corresponding motif profile needs to be provided. RNAMotifProfile identifies and separates the loop regions at the beginning. Each of the motifs is then converted into a profile of a single motif. RNAMotifProfile calculates the corresponding alignment score of each of these query motif instances’ profiles with the target profile and generates a sorted list of the input motif instances based on the alignment score in decreasing order. The instances with higher scores are supposed to be the instances of the provided motif family. Therefore, the top items of this list are the potential instances of the target motif family. Additionally, we calculate *z*-scores for each of the query profiles based on the profile-to-profile alignment statistics of the target profile with respect to all participating motif instances used to generate that profile. Obviously, the instances with higher alignment scores show higher *z*-scores and the instances with positive *z*-scores indicate that the instance is structurally closer to the target profile family. RNAMotifProfile supports scanning through multiple RNA chains to search for the instances of the provided motif profile. To achieve that, the name of the RNA chains needs to be provided in <PDBID><underscore><ChainID> format (e.g. 4V9F_0, 5TBW_1) as input to RNAMotifProfile while using the search mode.

Our approach for cutting the loop boundaries differed from the prior studies. We used a criterion of either one canonical base pair or two wobble base pairs to define the loop regions. This variation in loop-cutting criteria led to differences in the reported loop boundaries and lengths compared to the previous tools. For instance, through manual inspection, we identified five kink-turn motif instances within the 23S ribosomal RNA (rRNA) chain (PDB ID: 1S72, chain ‘0’), while previous studies using RNAMotifScanX and RNAMotifScan reported a larger number of kink-turn instances in this RNA. However, upon closer examination, we found that some of the previously reported instances (specifically, 1S72_0:21-26_517-522 and 1S72_0:794-798_815-819) exhibited similar base-pairing interactions to kink-turn, but did not fully conform to the characteristic kink-turn shape. Therefore, we excluded these instances from our manually curated kink-turn motif list.

Similarly, we found eight sarcin–ricin motif instances after manually analyzing all the internal loop motifs within the 23S rRNA chain (PDB ID: 1S72, chain ‘0’). Out of the identified sarcin–ricin motif instances by previous studies (RNAMotifScanX and RNAMotifScan), we decided to classify 1S72_0:952-956_1011-1015 motif as E-loop due to the 3D shape it exhibited in PyMOL.

## Results

### Collected dataset of well-known motif instances

We have used a dataset of 938 structural motifs that includes 358 internal loop motifs, 415 hairpin loop motifs and 165 multi-loop motifs. The internal loops are classified into 10 well-known structural motif families, while the hairpin loops fall into three such families. The locations of these motif instances along with their motif family annotation are collected from Ge *et al.* (21) and RNA 3D Motif Atlas (7) by following the same procedure described in RNAMotifContrast (24) paper. As there was no well-known motif family information for multi-loop motifs, we collected 46 clusters containing 165 multi-loop motif instances from the clustering result of Ge *et al.* (21) and considered the clusters as multi-loop motif families. The PDB chains of our input dataset are prepared following the Representative PDB list (32) release 3.320 at a resolution of 4.0 Å. The distribution of the motifs based on their count and average motif length along with minimum and maximum motif length for each family is provided in Table 1. The PDB locations of the internal loop, hairpin loop and multi-loop motifs are provided in Supplementary Tables S1, S2 and S3, respectively.

**Table 1.** The distribution of the internal and hairpin motif instances in well-known motif families and total number of multi-loops from 46 clusters used to test the performance of RNAMotifProfile

Loop type	Motif family	No. of motifs	Motif length, avg (min–max)
IL	Kink-turn (KT)	67	13 (11–16)
	Reverse Kink-turn (rKT)	8	25 (23–26)
	Sarcin–ricin (SR)	74	13 (10–21)
	C-loop (CL)	44	8 (6–12)
	E-loop (EL)	49	10 (7–13)
	Hook-turn (HT)	34	10 (9–14)
	Tandem-shear (TS)	45	8 (8–11)
	Tetraloop-receptor (TR)	19	8 (6–9)
	L1-complex (L1C)	6	14 (12–18)
	Rope-sling (RS)	12	10 (10–13)
HL	GNAA	234	6 (6–8)
	GNGA	64	6 (6–9)
	T-loop	117	9 (7–13)
ML	Multi-loops^a^	165	21 (9–78)

IL, internal loop; HL, hairpin loop; ML, multi-loop.

^a^These loops are not from a single family as there is no such information. These are the motif locations classified into 46 clusters collected from the work of Ge *et al.* (21), consisting of 105 three-way, 47 four-way and 13 five-way multi-loop motifs.

### Profiles built with RNAMotifProfile

We used RNAMotifProfile to build profiles for all 10 well-known internal loop motif families, 3 hairpin loop motif families and 46 multi-loop clusters provided in Table 1. RNAMotifProfile produces a text file containing the profile information for each input motif group as output. By using the representation criteria discussed in the ‘Materials and methods’ section, we created the profile images for all these output profiles. As mentioned before, the profile images exclude scarce base-pairing interactions and abundant gaps. However, the common base-pairing interactions and nucleotides are apparent in the profile images. Therefore, the profiles can be utilized to effectively analyze and identify characteristic base interactions of a motif family. Moreover, the profiles can also be useful in studying the structural variations in a motif family.

Out of the 10 internal loop motif families, the kink-turn and sarcin–ricin families have been more extensively studied and characterized in the existing literature. The wealth of prior research on these motif families provides a solid foundation for further in-depth investigation and facilitates a more comprehensive understanding of their structural properties and functional implications. Moreover, these two are the families with the highest number of instances (67 and 74, respectively) in our input dataset. Therefore, we choose to concentrate our analysis on these two families as a representative of internal loop motif families. Additionally, we have included the GNAA motif family profile from the hairpin loop motifs as it contains the highest number of instances (234). Furthermore, a multi-loop profile is also included that is built from a cluster of 11 three-way junction loops. The detailed analysis on these profiles is presented in the following subsections.

### Kink-turn motif family profile

Kink-turn (9) is one of the most well-known and well-studied motif families among all RNA motif structures. A regular kink-turn motif is an asymmetric internal loop consisting of a three-nucleotide bulge followed by G–A and A–G base pairs (34) and has a kink causing a sharp turn in the RNA helix (9). It also has a flanking C–G base pair on one side and a sheared G–A pair on the other side. The characteristic structure and high stability made it a key architectural element in RNA structures (34). The structure is also important due to its ability to act as a binding site for specific proteins (35). There is more than one study (36–39) that confirms three characteristic base-pairing interactions in a regular kink-turn structure. A tandem *trans*-H/S at the end of the NC stem and two crossing *trans*-sugar/sugar (*trans*-S/S) are the most significant base pairs for this motif family. Moreover, there could be another *trans*-S/H pair as well in the NC helix (39).

**Figure 4.**
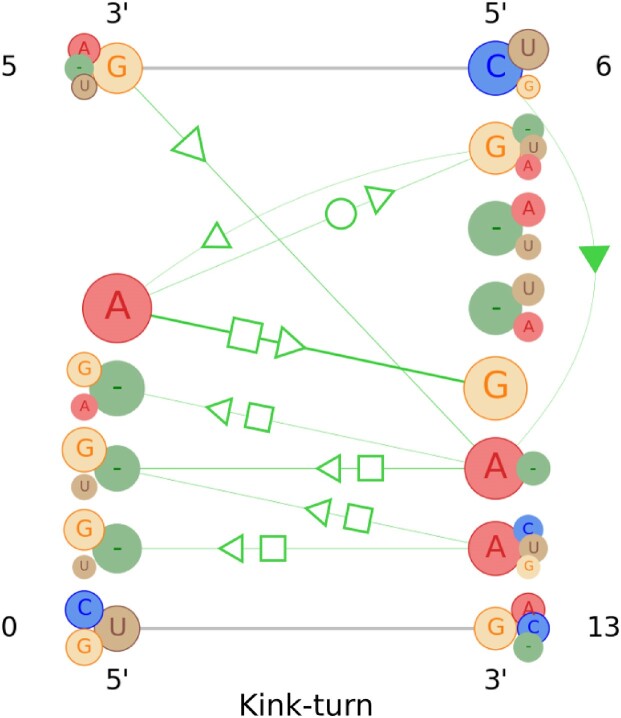
The visualization of the profile information gathered from the instances of the kink-turn motif family. Each nucleotide position can have four nucleotides or gaps based on their appearance in the kink-turn instances used. Their varying size represents the frequency as well as the significance of a particular nucleotide in a specific position. Larger circles represent the nucleotides with higher weight, and vice versa. Green lines with varying thicknesses represent the base pairs. The thicker lines depict the base pairs that are observed more. For each base pair, the circles representing the nucleotides involved in the interaction are highlighted with a circular outline. Circles without an outline indicate nucleotides that are present at that position but do not participate in the base-pairing interaction depicted. More specific details of the base interaction can be found in the text outputs of the *.pfl files.

We have collected 67 instances of kink-turn motifs and generated a profile based on their structures and alignments. The visualization image generated from the RNAMotifProfile output is provided in Figure 4, which shows almost all of these characteristic base pairs mentioned earlier. It contains the two-to-four nucleotide bulge as well as the two G–A and A–G base pairings. Moreover, the kink-turn profile image contains the characteristic tandem *trans*-H/S base pairing at the end of the NC stem and two crossing *trans*-sugar/sugar (*trans*-S/S) base pairs mentioned in more than one study. The edges representing the crossing *trans*-S/S base pairings in Figure 4 are thinner, which means although these interactions are part of the characteristic kink-turn structure, they might be missing in some of the kink-turn motif instances. Additionally, it is visible in Figure 4 that the appearance of the tandem *trans*-H/S might vary due to an extra nucleotide in the shorter strand.

### Sarcin–ricin motif family profile

Sarcin–ricin (10) is another well-known RNA structural motif family and also an asymmetric internal loop. This highly conserved structure is generally found in the RNA of all large ribosomal subunits (10,40) and is an essential site for RNA–protein interaction (41,42). Based on the research work described in (43), the appearance of a generic sarcin–ricin motif includes a *trans*-H/S, a *trans*-Watson–Crick/Hoogsteen (*trans*-W/H), a *cis-*Hoogsteen/sugar (*cis*-H/S), a *trans*-Hoogsteen/Hoogsteen (*trans*-H/H) and a *trans*-S/H base pair.

**Figure 5.**
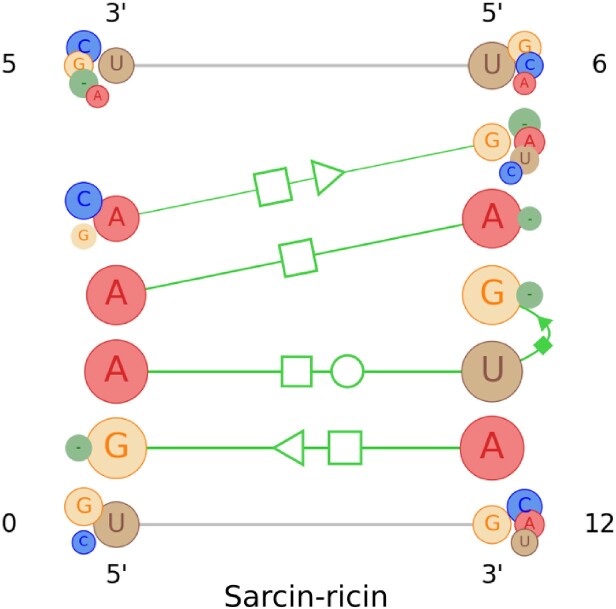
The visualization of the profile information collected from the instances of the sarcin–ricin motif family. As seen in Figure 4, larger circles represent the nucleotides with higher weight, and vice versa. Also, the base pairs are represented with green lines where the thicker lines depict the base pairs that are observed more. For each base pair, the circles representing the nucleotides involved in the interaction are highlighted with a circular outline.

We used a set of 74 motif instances of the sarcin–ricin family to generate the profile by employing our method. The visual representation of this profile is shown in Figure 5. It contains all five base pairs described above and the thicknesses of the edges representing these interactions are high, which means they are common in almost all the instances we have used. The G-bulge region along with the nearest *trans*-H/S and G/U/A base triplets with *cis*-S/H and *trans*-W/H seem to be the characteristic base pairs of the sarcin–ricin motif family. This claim also perfectly aligns with the description provided in (44). The profile outcomes for any group of motif instances might contain one or more extra base-pairing interactions that could be a variation that either was observed in a few of the input instances or appeared due to annotation or alignment inaccuracies.

### GNAA motif family profile

**Figure 6.**
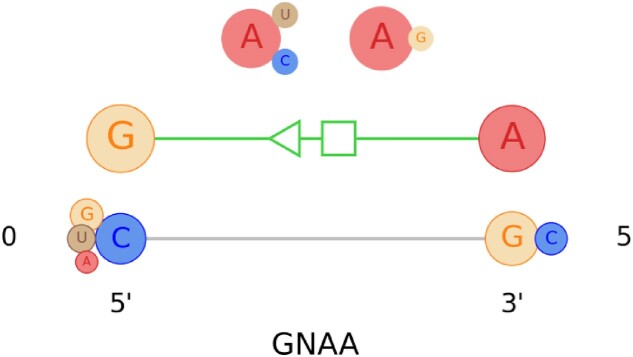
The visualization of the profile information for the GNAA motif family. As seen in Figure 4, larger circles represent the nucleotides with higher weight, and vice versa. Also, the base pairs are represented with green lines where the thicker lines depict the base pairs that are observed more. For each base pair, the circles representing the nucleotides involved in the interaction are highlighted with a circular outline.

GNAA (N is any nucleotide) is a pretty simple hairpin loop with mostly a *trans*-H/S base pair in the first and the last nucleotide base. However, this is a highly conserved hairpin loop motif in both sequence and structures. We had two other hairpin loop motifs (GNGA and T-loop) in our input dataset, but we choose to show the GNAA profile here due to having better number of instances (234 out of 415 hairpin loop motifs) for this motif. Figure 6 shows the visual representation of the GNAA motif profile. Although, we found *trans*-Hoogsteen/Watson-Crick (*trans*-H/W) pair for some instances (11 out of 234, which is only 4.7% of the total instances) in the same location of the previously mentioned *trans*-H/S base pair, the interaction is not shown in the figure due to being less than the 10% threshold for base interactions discussed earlier.

### Profile from a multi-loop motif cluster

RNAMotifProfile can build a profile from a group of multi-loop motifs as well. However, there was not sufficient information regarding multi-loop motif families. Therefore, we collected multi-loop motif clusters from the clustering result of Ge *et al.* (21) and used those clusters to build the profile of each cluster. In our current implementation, the generation of the multi-loop motif profile is contingent on the multi-loops being composed of junctions of the same type. This design choice was made to ensure the structural coherence and consistency of the profile, as multi-loops with heterogeneous junction types may exhibit different structural characteristics that could confound the analysis.

**Figure 7.**
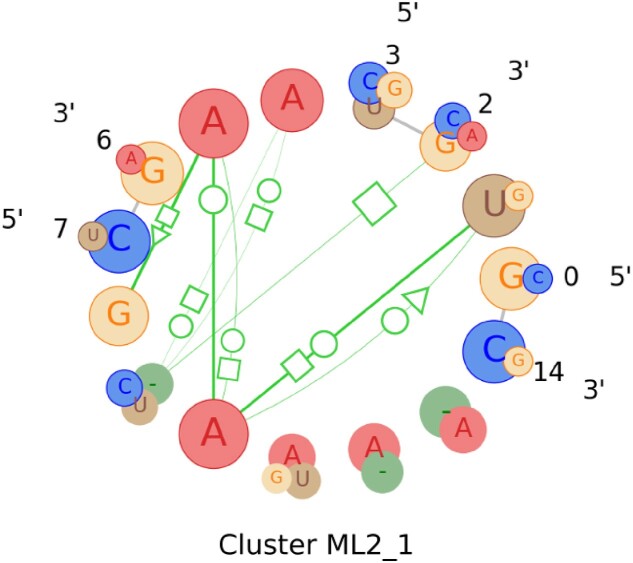
The visualization of the profile is built from a multi-loop motif cluster. As seen in Figure 4, larger circles represent the nucleotides with higher weight, and vice versa. Also, the base pairs are represented with green lines where the thicker lines depict the base pairs that are observed more. For each base pair, the circles representing the nucleotides involved in the interaction are highlighted with a circular outline. Due to having more than two junctions, the strands of the profile are drawn in a circular orientation.

In our collected clusters, there were 6 out of 46 clusters that are of mixed junction types. Therefore, we build the profiles for 40 clusters, those having the same types of junction loop motifs. Out of these 40 clusters, ML2_1 is a cluster of three-way junction loops containing 11 multi-loop motif instances. This is the highest number of instances we found from the collected clustering data. RNAMotifProfile successfully built the profile for this cluster from the 11 motif instances, and the visual representation of this profile is shown in Figure 7. Based on the figure, it appears that the majority of the instances in cluster ML2_1 feature a combination of three base pairs: a *trans*-H/S pair, a *trans*-H/W pair and a *trans*-Watson-Crick/Watson-Crick (*trans*-W/W) pair.

### Search results with profiles

RNAMotifProfile uses a profile-to-profile alignment algorithm to build the profile of a group of input motif instances (i.e. a motif family). This profile can then be utilized to search for unknown instances of the same motif family. In contrast, RNAMotifScanX or RNAMotifScan uses a manually pre-generated consensus structure to search for the instances of a motif family, while FR3D uses a single motif instance to search for similar instances. Therefore, if an unknown instance of a known motif family with structural variation is encountered, these tools may find inadequate matching base interactions, resulting in a low alignment score. As RNAMotifProfile automatically generates the profile incorporating all the variations provided in the input motif group, it has the potential to find a good number of matching base interactions. Still, the previous tools are able to provide higher specificity with respect to the profile-based search in limiting false positives. However, as shown in the following subsections, RNAMotifProfile definitely provides higher sensitivity in detecting authentic base pairings and variations.

To evaluate the performance of RNAMotifProfile against existing structural motif search tools, we used the 23S rRNA from *Haloarcula marismortui* (PDB ID: 1S72, chain ‘0’) as a benchmark. This RNA structure has been extensively studied and previously utilized by other tools.

Using RNAMotifProfile, we built a kink-turn profile from 67 known instances and a sarcin–ricin profile from 74 known instances. We then employed these profiles to search for occurrences of the respective motifs within the 23S rRNA chain. Using the search feature of RNAMotifProfile, with respect to the corresponding motif profile, we scan through the input RNA chain and provide a list of structural motif instances as output. The output includes profile-to-profile alignment score of each motif instance with respect to the input profile. The list is sorted based on the alignment score in descending order. Additionally, based on the mean and standard deviation of the alignment scores against the corresponding profile with all participating instances, we calculate the *z*-score of listed motif instances in the search result. The search results, along with a comparison to the outputs of RNAMotifScanX, RNAMotifScan and FR3D, are presented in the following subsections.

### Search results of the kink-turn motif family

To facilitate a direct comparison, we matched the kink-turn motif instances identified by RNAMotifProfile with those reported by RNAMotifScanX, RNAMotifScan and FR3D, based on the covered sequence regions. The compiled search results, including only those instances that generated a positive *z*-score, are presented in Table 2. RNAMotifScanX, RNAMotifScan and FR3D search ranks are provided in separate columns, while the manual annotation is added in the second to last column. After close inspection, we identified whether an instance is a regular one or a variation and this information is added in the last column.

**Table 2.** Top eight search results that have positive *z*-score while searching using kink-turn motif profile

				Rank with existing tools			Manual annotation	
Rank	Motif location	Score	*z*-score	RNAMotifScanX	RNAMotifScan	FR3D	Family name	Variety
1	1S72_0:1147-1154_1213-1216	122.27	2.56	4	4	3	Kink-turn	Regular
2	1S72_0:77-81_93-100	116.44	2.34	1	1	Q^c^	Kink-turn	Regular
3	1S72_0:1587-1592_1602-1608	86.32	1.2	-	8^a^	10^a^	Kink-turn	Regular
4	1S72_0:1312-1319_1338-1342	86.19	1.2	3	2	4	Kink-turn	Regular
5	1S72_0:937-940_1026-1033	78.68	0.91	2	3	2	Kink-turn	Variation
6	1S72_0:21-26_517-522	72.72	0.69	8^b^	-	-	E-loop	
7	1S72_0:794-798_815-819	72.72	0.69	6^b^	7^b^	-	E-loop	
8	1S72_0:1764-1769_1774-1780	72.56	0.68	-	-	-	Sarcin–ricin	

The ranking of the same motifs with existing tools are provided. Manually annotated family name and variety information are added in the last two columns.

^a^Ranked after unrelated motifs.

^b^Considered as instance from different family based on the 3D structure (although they share some of the interactions with kink-turn motif family).

^c^Q marks the query motif that was used to search through the RNA chain for similar structures using FR3D.

**Figure 8.**
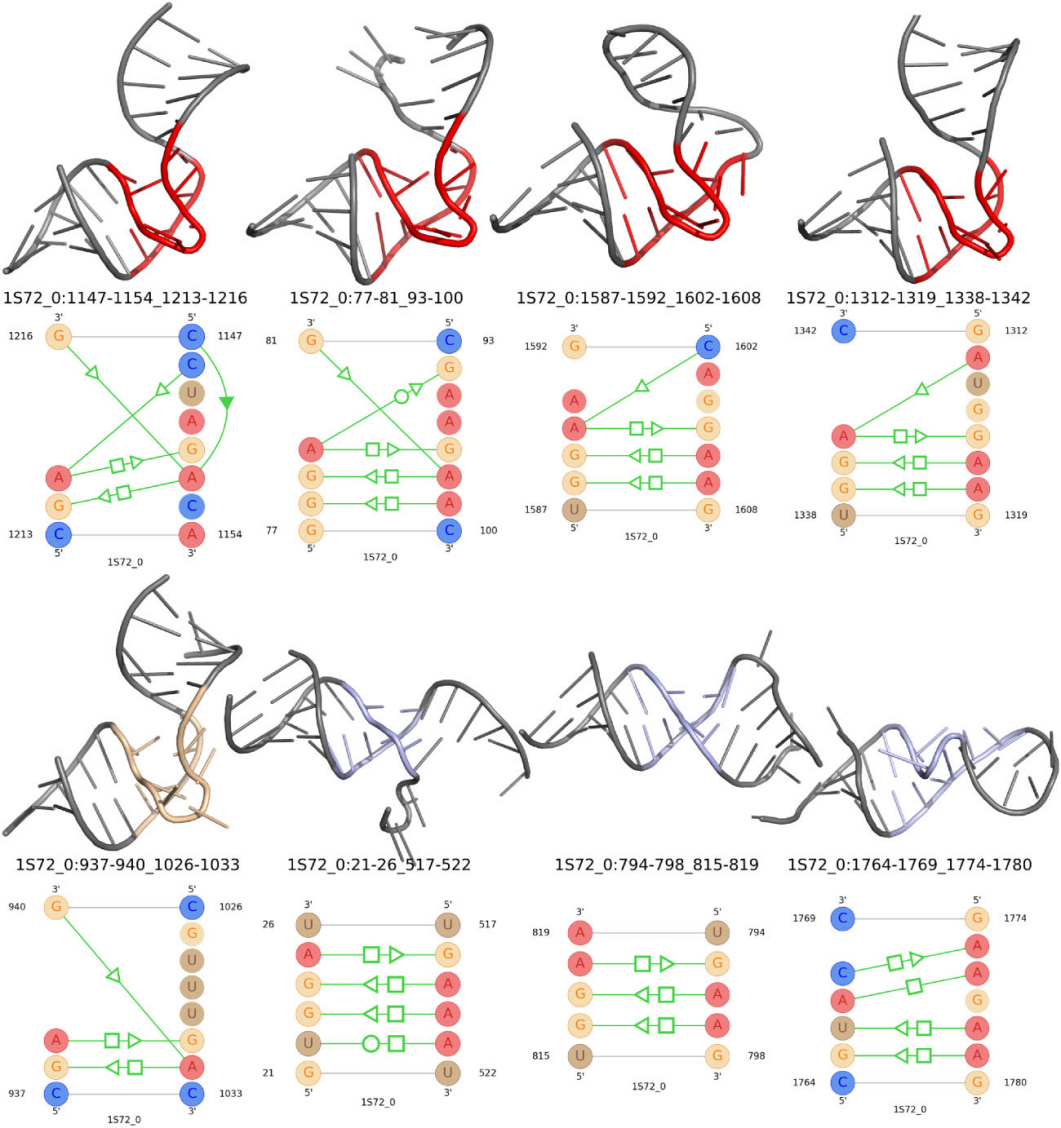
Top eight search results listed in Table 2 along with their base-pairing interactions. Regular instances are shown in red color, while the variation is represented using wheat color. The instances from unrelated motif family is shown in light blue color.

By utilizing the PDB data for the motifs listed in Table 2, we have generated their structural images using PyMOL, which is provided in Figure 8. The base-pairing interactions for these motifs are also added in the same figure. Regular kink-turn instances are shown in red color while the variation of the kink-turn motif is shown in wheat color. The manually identified non-kink-turn instances are represented with light blue color.

From the 3D structural images and their base-pairing interaction in Figure 8, the first four motif instances are the regular kink-turns with characteristic base-pairing interactions. The fifth one looks like a variation and the difference is also visible with a missing *trans*-S/S interaction. It also lacks of one *trans*-S/H interaction compared to the regular kink-turn motif interactions. The sixth to eighth motif instances do not have the kink-turn shape at all, although they share some of the base-pairing interactions. Due to the common base pairs, they produced a good alignment score with the kink-turn profile, which in turn shows a positive *z*-score. However, the instances are listed after the regular and variants of kink-turn motif instances in the search result provided in Table 2.

### Search results of the sarcin–ricin motif family

**Figure 9.**
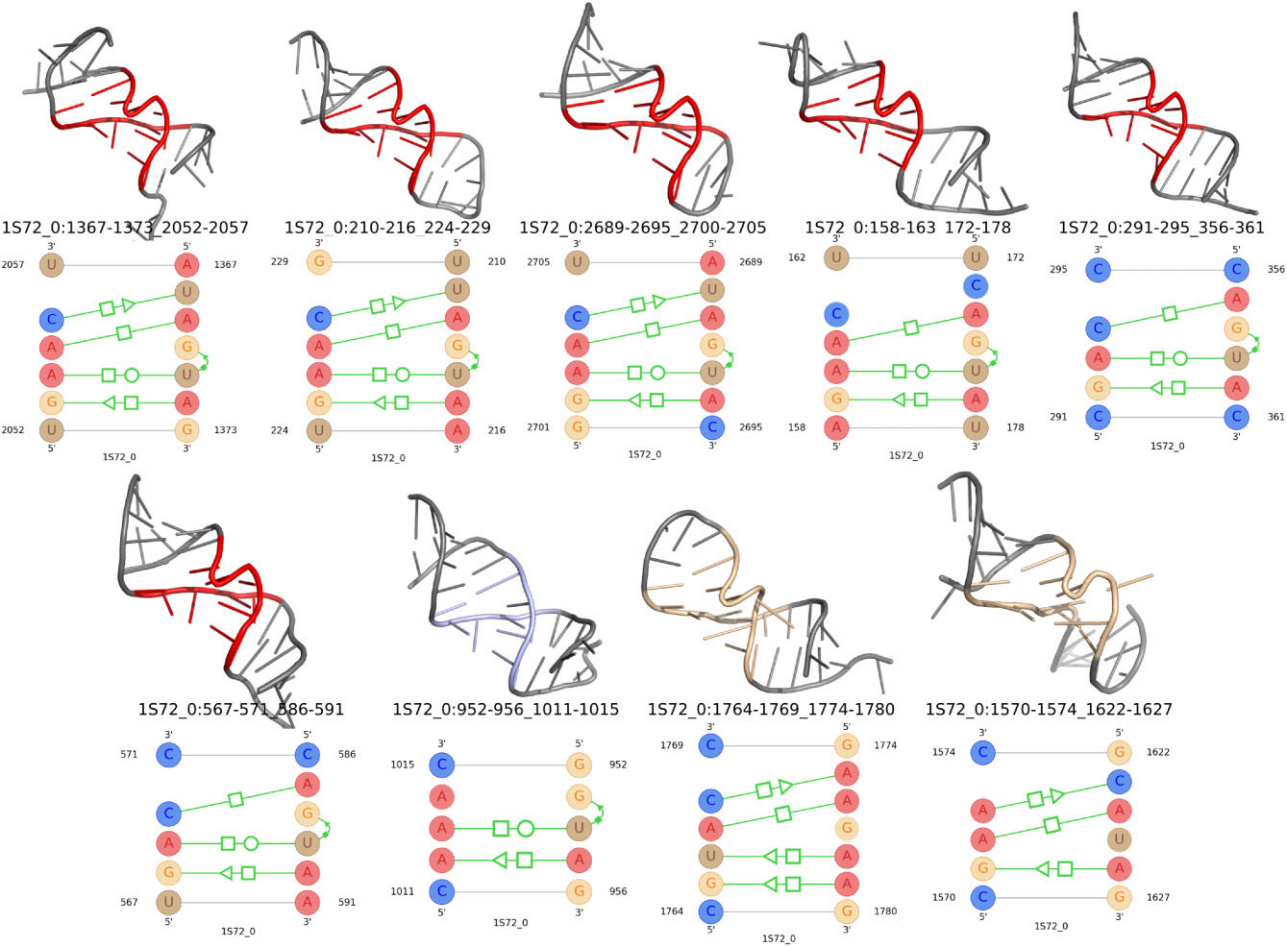
Top nine search results are listed in Table 3 along with their base-pairing interactions. Regular instances are shown in red color, while the variation is represented using wheat color. The instances from unrelated motif families are shown in light blue color.

We followed the same approach as kink-turn to search for the sarcin–ricin motif instances and the results containing the instances with positive *z*-score are presented in Table 3. Two manually identified sarcin–ricin instances were missed in this table due to their negative *z*-score. We have added these two instances at the end of the same table to show the performance comparison with previous tools. The images of the instances of Table 3 and images of their base-pairing interaction distribution are provided in Figure 9. The same color scheme for regular, variation and non-family instances is used in this figure as well.

**Table 3.** Top nine search results that have positive *z*-score while searching with sarcin–ricin motif profile

				Rank with existing tools			Manual annotation	
Rank	Motif location	Score	*z*-score	RNAMotifScanX	RNAMotifScan	FR3D	Family name	Variety
1	1S72_0:1367-1373_2052-2057	127.71	1.05	1	1	1	Sarcin–ricin	Regular
2	1S72_0:210-216_224-229	127.71	1.05	3	2	7	Sarcin–ricin	Regular
3	1S72_0:2689-2695_2700-2705	127.71	1.05	2	3	Q^c^	Sarcin–ricin	Regular
4	1S72_0:158-163_172-178	103.57	0.51	5	7^a^	4	Sarcin–ricin	Regular
5	1S72_0:291-295_356-361	103.23	0.5	10^a^	13^a^	6	Sarcin–ricin	Regular
6	1S72_0:567-571_586-591	103.23	0.5	9^a^	11^a^	3	Sarcin–ricin	Regular
7	1S72_0:952-956_1011-1015	79.28	−0.04	7^b^	8^b^	-	E-loop	
8	1S72_0:1764-1769_1774-1780	75.79	−0.12	16^a^	9^a^	-	Sarcin–ricin	Variation
9	1S72_0:1570-1574_1622-1627	73.37	−0.17	18^a^	-	-	Sarcin–ricin	Variation

The ranking of the same motifs with existing tools is provided. Manually annotated family name and variety information are added in the last two columns. The related instances with negative *z*-score are added in the bottom rows.

^a^Ranked after unrelated motifs.

^b^Considered as instances from different families based on the 3D structure (although they share some of the interactions with the sarcin-ricin motif family).

^c^Q marks the query motif that was used to search through the RNA chain for similar structures using FR3D.

In Figure 9, the first six instances are regular sarcin–ricin motif instances as they show the characteristic features in both 3D shape and base-pairing interactions. The seventh one shares three characteristic base-pairing interactions including the *cis*-sugar/Hoogsteen (*cis*-S/H) pair. However, the shape found using the PyMOL tool misses the S-shape. Therefore, we do not identify it as a sarcin–ricin. Due to the similar base-pairing interactions, it was reported as sarcin–ricin in previous tools (RNAMotifScanX, RNAMotifScan) and also it generated higher alignment score with the sarcin–ricin profile. The next two motifs contain a little bit different S-shape and most of the characteristic base-pairing interactions, but none of them has the *cis-*sugar/Hoogsteen (*cis*-H/S) pair. Additionally, the eighth instance has an extra *trans*-S/H pair in place of the *trans*-H/W base pair, while the ninth one does not have that interaction at all. Therefore, these instances generated a lower alignment score with the sarcin–ricin profile and had a negative *z*-score. Apart from that, due to the difference in their shape and base-pairing interactions, the last two instances were manually identified as a variation of the sarcin–ricin motif and RNAMotifProfile listed these instances shortly after the regular instances, while previous tools (RNAMotifScanX, RNAMotifScan and FR3D) listed them after more unrelated instances or did not list them at all.

### Computational efficiency of RNAMotifProfile

The profile-to-profile alignment algorithm discussed in this paper depends on the maximal optimal clique-finding algorithm. The runtime of the clique-finding algorithm solely depends on the size of the graph. The size of the graph will increase exponentially with the size of the input dataset. If a large number of motif instances are provided to generate their profile, they will have a higher number of interactions to compare, leading to a larger graph. For example, consider the comparison of two profiles A and B, where each of the profiles A and B was built from a single motif instance, A containing *p* interactions and B containing *q* interactions. The compatibility graph for aligning these profiles can have up to *pq* nodes. According to Tsukiyama *et al.* (45), a graph with *n* vertices can have at most 3^(*n*/3)^ maximal cliques. Thus, for a compatibility graph with *pq* vertices, there could be as many as 3^(*pq*/3)^ maximal cliques to evaluate. The alignment score calculation must be performed for each of these maximal cliques, which can be computationally expensive. The complexity further increases when a profile is built from multiple motif instances, as this can lead to more interactions and potential nucleotide choices at each position within the profile. To find the maximal cliques efficiently, we followed a recursive backtracking procedure listed by the Bron–Kerbosch algorithm (33).

RNAMotifProfile can build profiles considering both base-pairing and base-stacking interactions, but its runtime increases significantly for larger motifs due to the time complexity of the clique-finding algorithm. For instance, generating a profile from five kink-turn instances remained incomplete after 3 days of processing. The extended runtime is attributed to larger loops containing more base interactions, which expand the compatibility graph. This larger graph produces more candidates for the clique-finding algorithm, resulting in longer processing times. Our input dataset revealed an average of three base-pairing and nine base-stacking interactions in internal loop motifs, one base-pairing and five base-stacking interactions in hairpin loops, and five base-pairing and 14 base-stacking interactions in multi-loop motifs. The significantly higher number of base-stacking interactions compared to base pairs reduces the time efficiency of the clique-finding algorithm during profile building. To address this issue, we modified our approach to consider only base-pairing interactions for the clique-finding algorithm while still utilizing both base interactions for alignment score calculations. This modification substantially reduced runtime while maintaining profile quality. Experimental comparisons of hairpin loop motif family profiles, with and without considering base-stacking interactions during clique finding, showed no significant differences (Supplementary Figure S1). With this optimization, generating a profile for 67 kink-turn motif instances takes approximately 105 s (averaged from five runs), assuming all related data (PDB and FASTA files, loop file, annotation data and partial PDB data) are pregenerated. Tests were conducted on a system with a 3.20 GHz × 12 Core™ i7-8700 CPU and 64 GB of memory, running Ubuntu 18.04.6 LTS. Superior hardware specifications would likely further improve runtime. For users who prefer to include base-stacking interactions in clique finding, this option remains available by modifying the *get_include_stackings_flag* function in the *config.py* file when running RNAMotifProfile. The implementation leverages Python multiprocessing to maintain a moderately low overall runtime, though further optimizations could potentially enhance performance.

## Discussion and conclusion

In this paper, we have presented a novel method to automatically generate RNA structural profiles for RNA motifs. The method takes a set of input motif instances and generates comprehensive profile data, which is then utilized to create graphical representations showcasing the key interactions. The visualization images prepared in this work aim to provide a clear and informative depiction of the motif structures. To maintain clarity, the figures do not display base-pairing interactions that appear in less than 10% of the input instances. Similarly, nucleotides that appear in less than 10% of the input instances are omitted from the visualizations. Additionally, gap positions introduced in more than 75% of the motif instances are omitted if they are not associated with important base interactions. All base-stacking interactions are also excluded from the visualizations, though these data are retained in the generated profile. These filtering choices were made to keep the visualizations clean and easy to interpret. The same filtering criteria are applied while searching using a profile. However, the intensity of filtering can be adjusted by modifying the command line parameters while compiling the source code of RNAMotifProfile. The full profile data, including the filtered information, remain available for downstream analysis and applications. The structural profiles generated by RNAMotifProfile can offer valuable insights into the similarities and variations within a motif family, compared to existing resources. Additionally, these profiles can be utilized to search for similar motif instances, even when accounting for structural variations, facilitating a deeper understanding of motif families and their potential functional implications.

In our implementation, we have leveraged the base interaction annotations from DSSR (30) and FR3D (19) tools. While these are reliable sources, any performance issues or inaccuracies in these external tools could affect the quality of the RNAMotifProfile results. We have benchmarked the performance of RNAMotifProfile as a motif search tool and found it to have good sensitivity, capable of detecting potential motif variations. However, the specificity could be lower compared to manually curated consensus structures, which may lead to some false positive results.

Overall, RNAMotifProfile provides a valuable tool for obtaining a holistic view of RNA motif structures, incorporating similarities and variations within a motif family. The method offers numerous customization options to adjust the output based on user requirements, such as filtering gaps, using branch-and-bound techniques and fine-tuning the alignment scoring parameters. This work presents a significant advancement in the field of RNA structural analysis and motif characterization.

## Supplementary Material

lqae128_Supplemental_Files

## Data Availability

RNAMotifProfile source code is publicly available on GitHub (https://github.com/ucfcbb/RNAMotifProfile) and Zenodo (https://doi.org/10.5281/zenodo.13625899).
