# Supplementary material for: RNAMotifProfile: a graph-based approach to build RNA structural motif profiles
Source: NAR Genom Bioinform. 2024 Sep 26;6(3):lqae128. doi: 10.1093/nargab/lqae128 (PMC11426329; doi:10.1093/nargab/lqae128)
Supplement: lqae128_Supplemental_Files [file lqae128_supplemental_files.zip › Supplementary_Materials.pdf]

Supplementary Materials for "RNAMotifProfile: a graph-based approach to build RNA structural motif profiles"

Md Mahfuzur Rahaman and Shaojie Zhang*

*To whom correspondence should be addressed. Email: shzhang@cs.ucf.edu

Algorithm S1 Build profile from N instances

Ensure: $Profiles \Leftarrow$ the list of one-instance profiles
Ensure: $N \Leftarrow$ the size of $Profiles$
Ensure: $K \Leftarrow$ the number of items in each sub-list

```
function BuildProfile(Profiles, N, K)
    if N = 1 then
        return Profiles[1] // return the first element
    end if
    if N > K then
        M = ⌈ N/K ⌉
        Create M sub-lists each containing K items
        Initialize a temporary profile list T
        for each sub-list S in M do
            P = BuildProfile(S, K, K)
            Add P to T
        end for
        return BuildProfile(T, M, K)
    end if
    i, j = FindBestProfilePairIndices(Profiles)
    Merge i-th and j-th profile, and update Profiles list
    //The list contains N − 1 items now
    return BuildProfile(Profiles, N − 1, K)
end function

function FindBestProfilePairIndices(Profiles)
    max_score = −Inf
    for i = 1 to n − 1 do
        for j = i to n do
            clique = maximal optimal clique
            between i-th and j-th Profile
            considering all profile combinations
            based on junction count
            score = corresponding alignment score
            if score > max_score then
                Update max_score
            end if
        end for
    end for
    return Index pair (i, j) for which max_score is found
end function
```

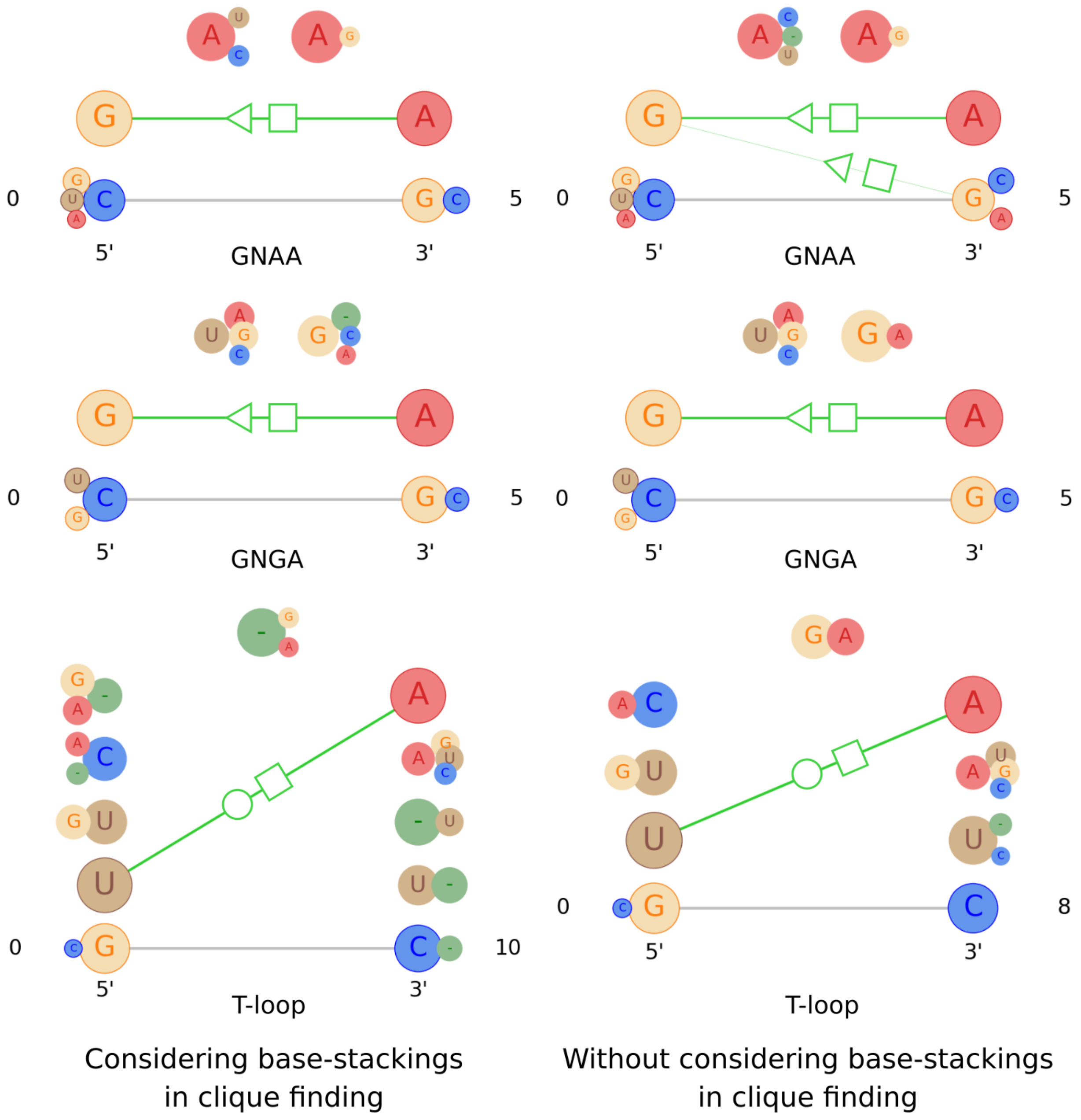

Figure S1: Comparison of three Hairpin loop profiles with and without considering base-stacking interactions in clique finding while aligning the instances. The profiles in the left column are produced without considering base-stackings while the profiles in the right column are produced considering them.
